# Delayed Hypoxic Reversible Leukoencephalopathy Syndrome

**DOI:** 10.7759/cureus.2702

**Published:** 2018-05-29

**Authors:** Anudeep Yelam, Pradeep C Bollu

**Affiliations:** 1 Department of Neurology, University of Missouri, Columbia, USA

**Keywords:** anoxic encephalopathy, white matter changes, demyelination, leukocyte arylsulfatase a

## Abstract

We report a case of postanoxic leukoencephalopathy in a patient who started to have cognitive and behavioral changes weeks after the anoxic insult along with white matter lesions on neuroimaging and demyelination on brain biopsy. His disease course followed a steady decline initially both clinically and radiologically and assumed a steady plateau. Months after his decline, the patient was seen to be completely functional with substantially improved mental status examination and resolution of white matter changes on imaging. The course of this disease entity usually assumes a plateau after clinical worsening with little improvement subsequently. However, our patient showed a dramatic recovery to his baseline after a few months. In this article, we review mechanisms, presentation and the sequelae of hypoxic injury to the brain.

## Introduction

Delayed hypoxic reversible leukoencephalopathy syndrome (DHRLS) is a rare entity characterized by neurological relapse after initial recovery following an acute hypoxic insult. Prolonged hypoxia leads to impaired oxygen delivery which causes disruption of enzymatic pathways involved in myelin turnover, resulting in delayed demyelination. Diagnosis is by history, clinical course and neuroimaging. Treatment is supportive.

## Case presentation

Mr. R is a 36-year-old right-handed male who was admitted to Neurology service for his altered mental status. The patient has a history of hypertension, fibromyalgia, obstructive sleep apnea, depression and substance abuse. One month prior to his presentation, he underwent cardiopulmonary resuscitation (CPR) when he was found unresponsive at his home. The CPR reportedly lasted for 3-4 minutes. He was hospitalized and was found to be in respiratory failure and acute renal failure. He was intubated and was treated for his renal failure. He gradually recovered and was discharged. His family members started to notice gradually worsening behavioral changes and short-term memory problems because of which, he was brought back to the hospital again.

**Figure 1:**
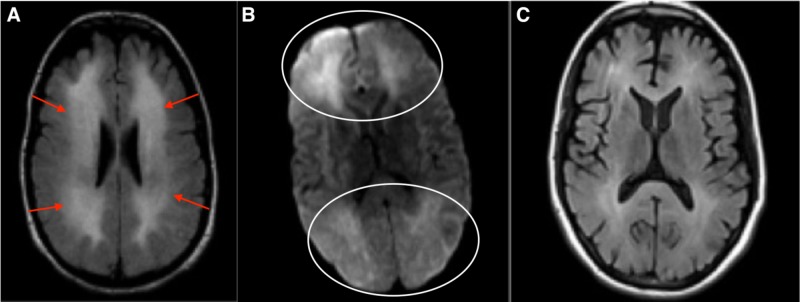
A. FLAIR, B. Diffusion, C. Follow-up MRI after one year. (A) shows increased FLAIR signal in the cerebral white matter (arrows). (B) shows diffusion restriction both anteriorly and posteriorly (oval circles). (C) shows improvement in the T2/FLAIR hyperintensities one year after the initial brain imaging. FLAIR: Fluid-attenuated inversion recovery; MRI: Magnetic resonance imaging.

During the course of hospitalization, the patient showed gradual deterioration in his mental faculties. He underwent two magnetic resonance imaging (MRI) studies – first one during the initial admission and the second one, 10 days later. The initial MRI showed T2/FLAIR hyperintensity signal involving the cerebral white matter. The repeat MRI showed significant worsening of the T2 Flair hyperintensities in white matter (Figures 1A, 1B). Neuropsychiatric evaluation showed that the patient showed significant deficits in his mental faculties including judgment and memory. A battery of blood and cerebrospinal fluid (CSF) studies including complete blood count (CBC), thyroid function studies, vitamin B12, human immunodeficiency virus (HIV), hepatitis panel and paraneoplastic antibody testing was ordered for the evaluation of his rapidly progressive cognitive decline and none of them came out to be abnormal. The patient later underwent a single-photon emission computed tomography (SPECT) scan followed by brain biopsy, which showed an area of demyelination. Post-biopsy, the patient was administered a trial of steroids which did not change his course. By this time, the patient’s cognitive decline came to a halt and was discharged to a nursing home. It was thought that the initial anoxic injury was responsible for his cognitive decline. Patient’s follow-up visit after two months showed a significant improvement in his mental faculties along with near complete resolution of the white matter changes (Figure 1C). An Institutional Review Board (IRB) approval from the University of Missouri was obtained for the publication of this case report. The IRB approval number for this case is 2011511.

## Discussion

Cerebral white matter is a target of hypoxic-ischemic injury throughout life, in clinical settings ranging from periventricular leukomalacia in the neonatal period, cerebrovascular insults in adults, to vascular dementia in the aging brain. Traditionally it was thought that the grey matter is more prone to anoxic injury [1] but studies suggested that the white matter is more sensitive to ischemia [2]. During the periods of hypoxia/anoxia, the grey matter is able to maintain adequate perfusion because it has better vascular supply compared to white matter, which has widely spaced linear arterioles and fewer anastomoses making it vulnerable to the insult. Disruption of the central conducting pathways in white matter produces both cognitive deficits [3, 4] and various neurobehavioral syndromes. Hypoxic/anoxic insult to the brain can present in a variety of ways depending upon the extent of insult (Table 1).

**Table 1:** Degree of anoxic insults and symptoms.

Degree of insult	Mild hypoxia/anoxia	Severe anoxia	Post-anoxic sequelae
Symptoms	Inattentiveness Loss of concentration Poor judgment Motor incoordination without any change in the level of alertness	Brain death syndrome	Persistent vegetative state Permanent coma Amnestic state Extrapyramidal syndrome with or without dementia Choreoathetosis Cerebellar ataxia Myoclonus Tremor

An interesting and not so fully explained entity – ‘Delayed Post anoxic encephalopathy' [5, 6] – happens in a few individuals where there is an acute phase of neurological deficit that improves almost completely followed by a lucid interval averaging 1-4 weeks after which patients commonly develop neuropsychiatric problems (apathy, confusion, irritability, and occasionally agitation or mania). Many patients with this condition are mistaken for suffering from psychiatric disturbances [7].

**Figure 2:**
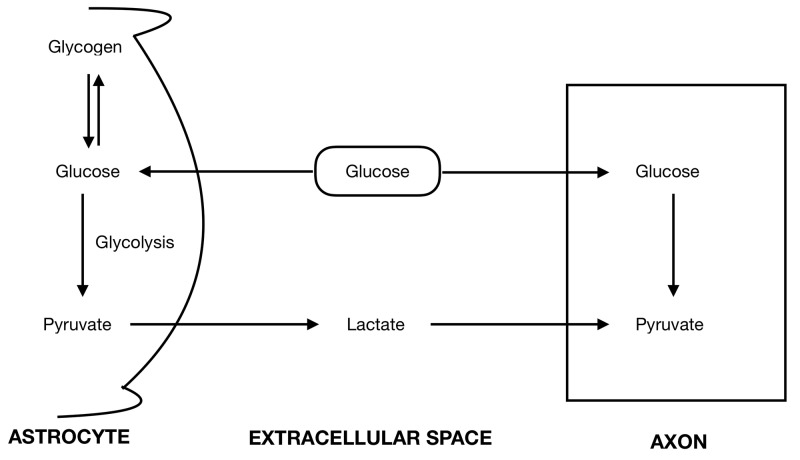
Schematic shows the interaction of astrocytes and axons with each other in regards to energy metabolism. Only astrocytes contain glycogen which is converted to lactate and transported to extracellular space. Axons take up the extracellular lactate and metabolize it to meet its energy requirements.

The mechanism of postanoxic leukoencephalopathy has not been fully understood. In the absence of glucose, central nervous system (CNS) axonal function can be met by intrinsic energy reserves provided through the breakdown of astrocytic glycogen [8] as shown in Figure 2. This mechanism operates for about 30 minutes [9] and the period of protection depends on the astrocyte glycogen content.

**Figure 3:**
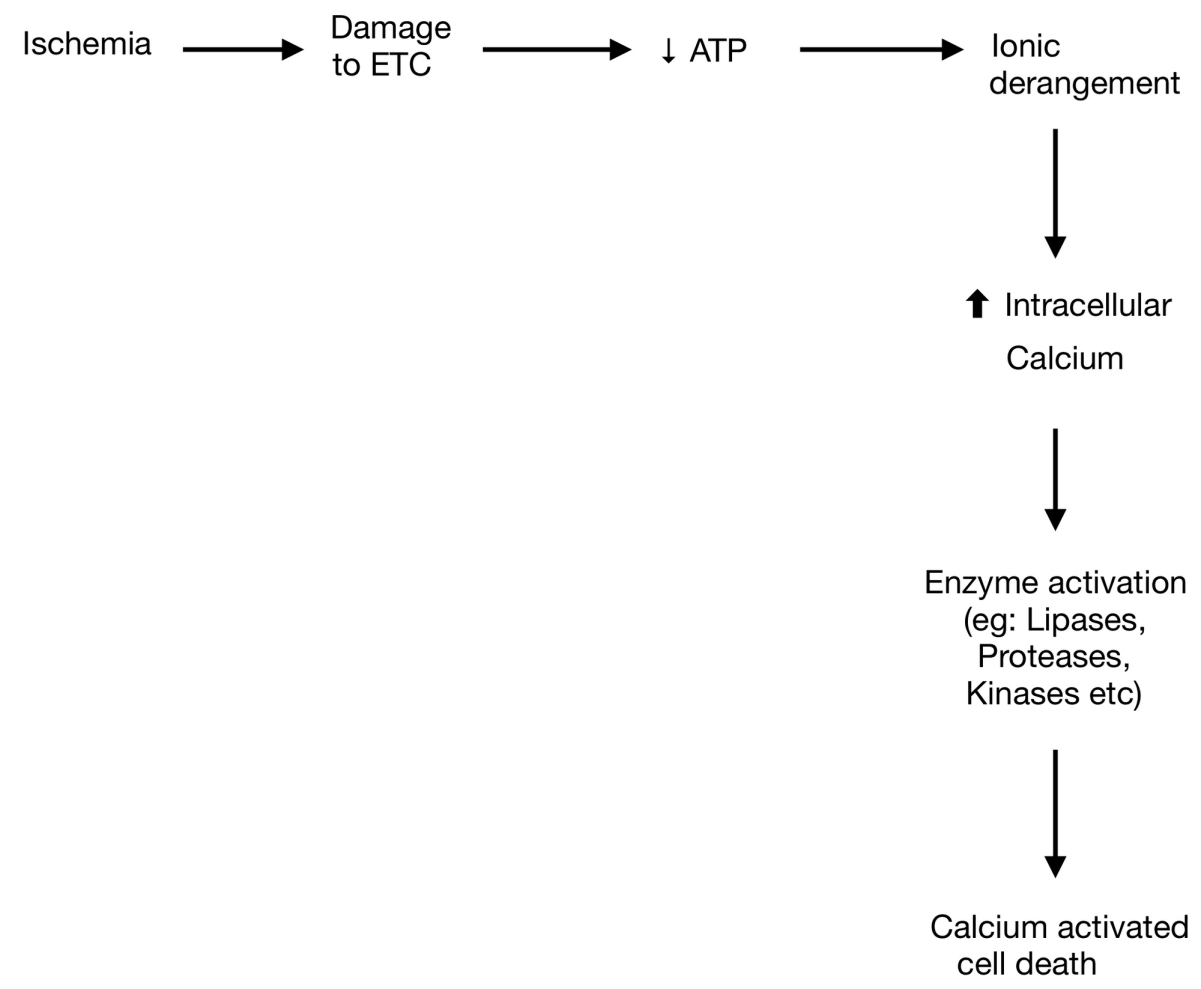
Schematic outlines the brain cell injury due to decreased energy supply. ETC: Electron transport chain; ATP: Adenosine triphosphate.

Studies using rodent optic nerves have shown that when there is energy deprivation, sodium and calcium channels are activated resulting in accumulation of intracellular calcium, which in turn leads to activation of multiple disruptive pathways [10] as shown in Figure 3.

**Figure 4:**
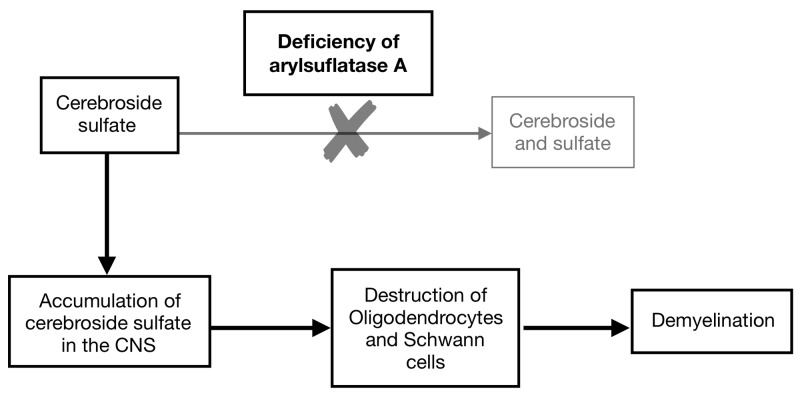
Schematic showing accumulation of sulfatides causing demyelination due to deficiency of arylsulfatase A. CNS: Central nervous system

Weinberger et al. [11] in their case report described a 34-year-old man who developed post anoxic demyelination after a drug overdose and was shown to have a reduction of arylsulfatase A (ARSA) activity to 10% to 30% of normal values. Gottfried et al. [12] also described similar findings in a patient who had a reduction of ARSA to approximately 50% of normal. Complete or incomplete deficiencies of arylsulfatase enzyme activity can predispose the development of post-hypoxic leukoencephalopathy. Interestingly, our patient did not have ARSA deficiency. Arylsulfatase A (ARSA), a lysosomal enzyme, is responsible for hydrolysis of cerebroside sulfate, a major glycolipid of myelin. So, deficiency of ARSA leads to accumulation of cerebroside sulfate in the central and peripheral nervous system thus causing the destruction of oligodendrocytes and Schwann cells. The end result is central and peripheral demyelination (Figure 4).

There are no widely accepted criteria for the diagnosis of delayed post anoxic leukoencephalopathy. Secondary causes of delirium and dementia should be ruled out before making the diagnosis of DHRLS. The history of hypoxic/anoxic episode, clinical course and neuroimaging features aids in the diagnosis of this condition. MRI findings are nearly pathognomonic and help in differentiating acute ischemic hypoxic encephalopathy, which shows early grey matter changes versus delayed postanoxic leukoencephalopathy, periventricular white matter changes with grey matter sparing [11] are seen. Areas of demyelination are also evident on brain biopsy. Myelin protein, a constituent of myelin, was also found to be elevated in patients with delayed hypoxic leukoencephalopathy [13].

There is no specific therapy but supportive care along with early rehabilitation has shown improvement in patients with this condition [14].

## Conclusions

A time delay between hypoxic/anoxic insult and deterioration along with a myriad of presenting neurologic symptoms make this diagnosis difficult. Most of the patients who sustain anoxic insult to the brain have poor functional outcomes with significant residual neurological disabilities. However, there are reports that some patients showed improvement with rehabilitation. In our patient, his cognitive and behavioral deterioration came to a halt and gradually showed a significant improvement over time.
